# Supplementary figures and images for: Bacterial pathogens deliver water/solute-permeable channels as a virulence strategy
Source: bioRxiv. 2023 Jul 29:2023.07.29.547699. Preprint. [Version 1] doi: 10.1101/2023.07.29.547699 (PMC10402153; doi:10.1101/2023.07.29.547699)

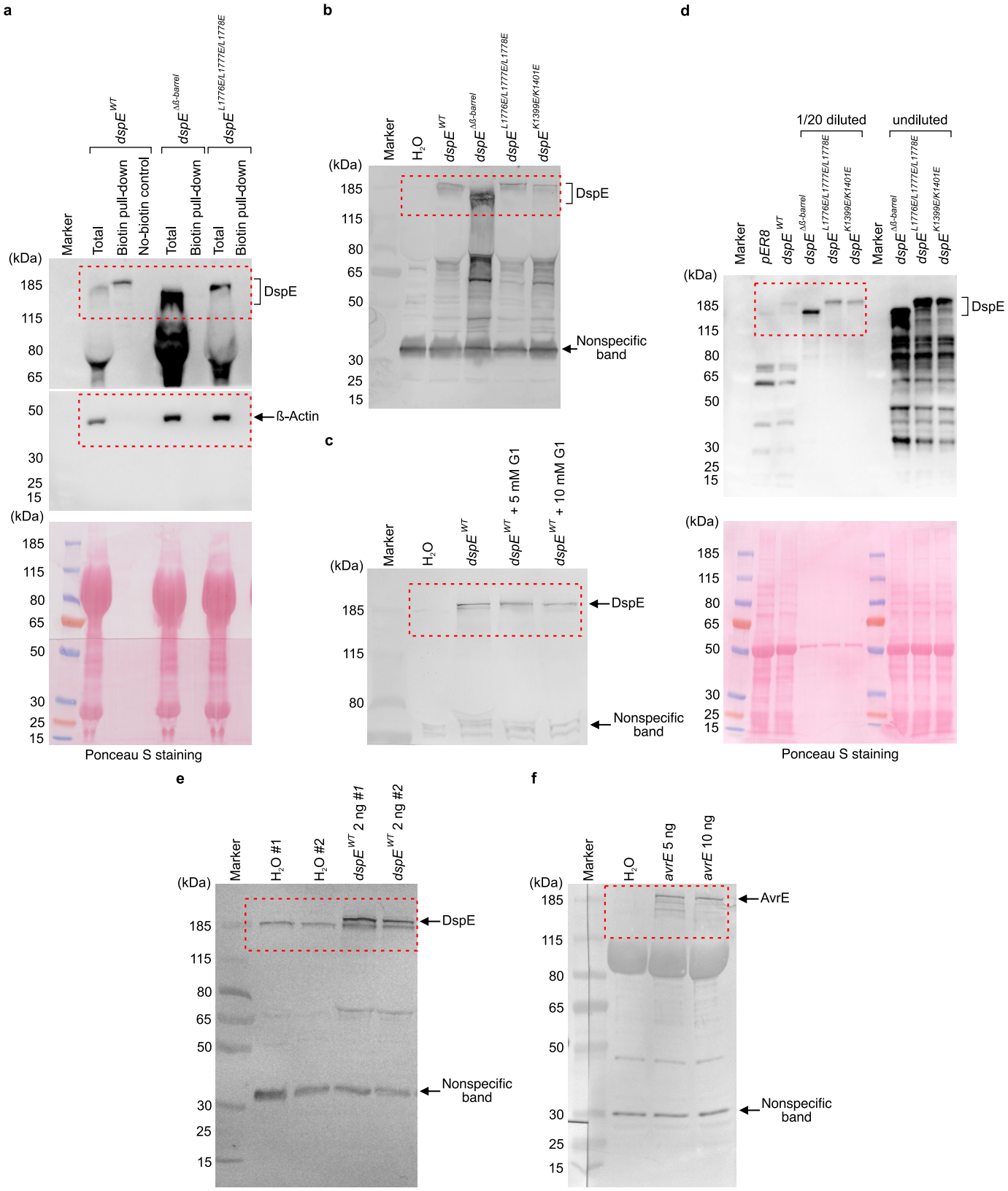

Supplement: Supplement 1 — Supplementary Figure 1. Whole gel images for Extended Data Fig. 4a–f. Dotted boxes show image cropping. [file media-1.pdf]

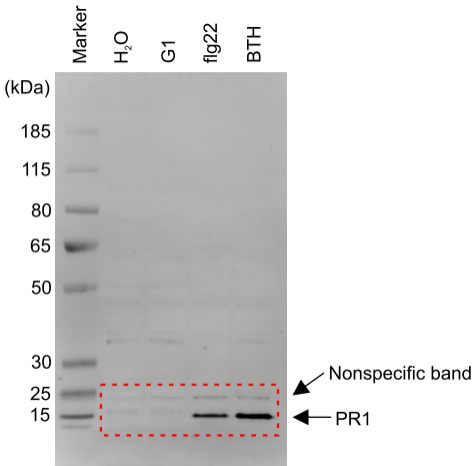

Supplement: Supplement 2 — Supplementary Figure 2. Whole gel images for Fig. 4c. Dotted boxes show image cropping. [file media-2.pdf]

**a**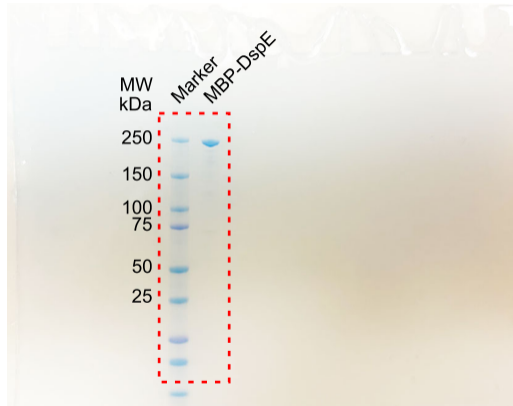**b**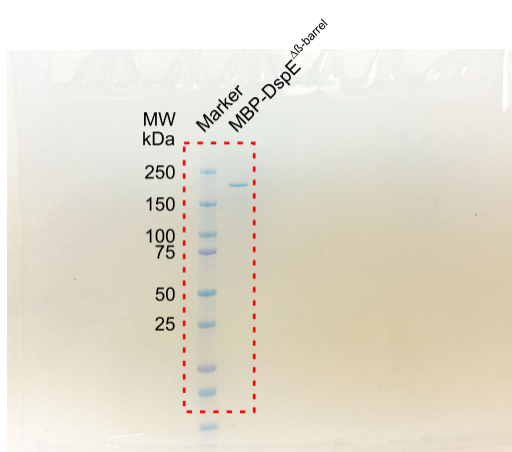

Supplement: Supplement 3 — Supplementary Figure 3. Whole gel images for Extended Data Fig. 4g,h. Dotted boxes show image cropping. [file media-3.pdf]
